# Diatom-Based Artificial Anode—Uniform Coating of Intrinsic Carbon to Enhance Lithium Storage

**DOI:** 10.3390/ma17184473

**Published:** 2024-09-12

**Authors:** Junlong Luo, Jun Cai, De Gong, Aoping Guo, Jaw-Kai Wang, Jiangtao Zhang

**Affiliations:** 1 School of Mechanical Engineering and Automation, Beihang University, Beijing 100191, China; luojl@buaa.edu.cn; 2 College of Chemistry, Beijing Normal University, Beijing 100083, China; 3 Shenzhan Jawkai Bioengineering R&D Center Co., Ltd., Shenzhen 518055, China

**Keywords:** silicon oxide anode, carbon, diatom, lithium storage, TG-FTIR-GCMS, DFT calculations

## Abstract

Pursuing improved electrode materials is essential for addressing the challenges associated with large-scale Li-ion battery applications. Specifically, silicon oxide (SiO_x_) has emerged as a promising alternative to graphite anodes, despite issues related to volume expansion and rapid capacity degradation. In this study, we synthesized carbon-coated SiO_x_ using diatom biomass derived from artificially cultured diatoms. However, the inherent carbon content from diatoms poses a significant challenge for the electrochemical performance of diatom-based anodes in large-scale applications. Subsequently, we conducted further research and demonstrated excellent performance with a carbon content of 33 wt.% as anodes. Additionally, real-time characterization of the carbonization process was achieved using thermogravimetry coupled with infrared spectroscopy and gas chromatography mass spectrometry (TG-FTIR-GCMS), revealing the emission of CO and C_3_O_2_ during carbonization. Furthermore, electrochemical tests of the processed diatom and carbon (PD@C) anode exhibited outstanding rate capability (~500 mAh g^−1^ at 2 A g^−1^), high initial Coulomb efficiency (76.95%), and a D_Li_^+^ diffusion rate of 1.03 × 10^−12^ cm^2^ s^−1^. Moreover, structural characterization techniques such as HRTEM-SAED were employed, along with DFT calculations, to demonstrate that the lithium storage process involves not only reversible transport in Li_2_Si_2_O_5_ and Li_22_Si_5_, but also physical adsorption between the PD and C layers. Exploring the integration of diatom frustules with the intrinsic carbon content in the fabrication of battery anodes may contribute to a deeper understanding of the mechanisms behind their successful application.

## 1. Introduction

During past decades, the urgent demand for high performance and renewable electrode materials has attracted many researchers [1,2,3]. Thereon, researchers attach importance to natural biomaterials [4,5,6] instead of artificial materials, which can reduce negative effects on the environment and human health. Moreover, due to the unique biomolecular assembly technology, many biomaterials have multiple functions and characteristics [7,8]. As the most representative examples, diatoms are a type of unicellular algae with more than 200,000 species. They have silica shells with various morphologies of circular, elliptical, square, or triangular, ranging from 2 to 200 μm in size [9]. Currently, energy storage materials derived from diatom synthesis primarily utilize diatomite. For instance, diatom shells serve as template materials for silicon-based material precursors [10]. Research involving diatomite includes its application in lithium ion batteries [11], lithium sulfur batteries [12], and polymer solar batteries [13]. The primary utilization of diatomite in the field of energy storage involves either dissolving the original substance or converting it into silicon-based materials, which is predominantly associated with thermal energy storage (TES) [14]. The utilization of natural diatoms in the realm of functional materials is illustrated in Table S1.

After completing the large-scale cultivation of diatoms in our research, we investigated the potential use of cultivated diatoms as Si-based anode materials. Silicon (Si) stands out as a promising anode material due to having the highest theoretical capacity (4200 mAh g^−1^), a low discharge plateau (<0.5 V), rich resources, and eco-friendly features. However, poor intrinsic conductivity, volume expansion, and unstable interfaces in lithiation, greatly hinder their practical applications for LIBs [15,16]. In this case, a SiO_x_ (0 < x < 2) anode has been intensively verified as a promising alternative for Si due to its enhanced volumetric stability (~100% expansion), high capacity (SiO: 2680 mAh g^−1^), and also industrial scalable availability [17,18,19]. To date, tremendous modification methods have been applied to optimize Si-based anodes, including microstructure construction [20,21,22] and binder redesign [23]. In addition, researchers have also conducted extensive research on electrolyte and battery management parameters [24,25].

In the case of LIBs, the 3D multistage porous structure of diatom can effectively disperse stress, thereby mitigating material damage from volume changes during charge and discharge. We have demonstrated that the diatom-based anode could achieve a high discharge capacity of 924 mAh g^−1^, with a coulomb efficiency reaching almost 100% at 400 mA g^−1^ after 200 cycles [26]. Meanwhile, an initial Coulomb efficiency (ICE) of more than 75% was obtained from the first capacity of 1349 mAh g^−1^ (discharge) to the following capacity of 1066 mAh g^−1^ (charge). ICE is an important parameter for the application of silicon-based anodes. The majority of reported ICE values are below 70%, with a significant portion hovering around 50%. The electrochemical properties can be compared in Table S2. In addition, researchers used FIB-SEM to observe a cycled si-based electrode; the diatom frustules were able to maintain a stable structure after cycling, which is attributed to their unique structure [27]. However, it still remains a main challenge to the large-scale application of diatom-based anode due to the lack of essential exploration of the specific mechanism in the process.

In this study, we aim to develop an industrial process for functionalized diatom anodes using large-scale artificially cultured diatoms (including Chaetoceros, Navicula, and Stephanodiscus). We propose a comprehensive approach to investigate the working mechanism of different carbon contents in diatom biosilica-based anodes through a combination of characterization techniques and theoretical calculations. Firstly, two powerful physical organic analytical techniques, TG-FTIR-GCMS and Raman characterizations, were employed to investigate the carbon layer of inactivated PDMs (pure diatom materials). In this case, TG-FTIR-GCMS simultaneous analysis was used to study thermal decomposition of the PDMs in real time, and TG–DSC was conducted as well. Following carbonization, the samples exhibited a composition of silica and carbonaceous components, retaining the layered porous structure of diatom frustules (sample PD@C: processed diatom and carbon). Furthermore, the mechanism of diatom-based cells was revealed via measuring the ionic conductivity and electrochemical properties of the PD@C anode. Additionally, DFT calculations demonstrated that lithium storage of the PD@C anode in part attributed to physical adsorption between the PD and C layer as well as effective physisorption, providing sufficient electrical contact sites. Therefore, this study provides fundamental understanding and mechanism analysis of diatom biomass for practical applications in lithium anodes.

## 2. Experimental Section

### 2.1. Materials

Inactivated raw diatoms were supplied by Jawkai Bioengineering Research and Development Center Company Limited, Shenzhen, China. Hydrochloric acid solution (1 M) and absolute ethanol were obtained from Aladdin. PVDF and Supper P were supplied by Shenzhen WeiDiFei Company Limited, Shenzhen, China. Chemicals were utilized as original received.

### 2.2. Synthesis of Composites

PDMs: Raw inactivated diatoms were washed with HCl (1 M), absolute ethanol, and deionized water several times until the liquid became colorless and transparent and then dried in a vacuum oven at 80 °C for 12 h.

Different carbon content of PD@C:

PD@C (i.e., PD@C-B) was obtained by heating PDMs for 3 h under 650 °C in an atmosphere of argon;

PD@C-A was obtained by heating PDMs for 3 h under 650 °C in an atmosphere of air;

PD@C-C_10_ (C_20_, C_30_) was obtained by heating PDMs for 10 min (20, 30 min) in an atmosphere of air and then for 3 h under 650 °C in an atmosphere of argon;

PD@C-D: PDMs and soluble starch (mass ratio = 3:1) were put into deionized water, mixed, and ultrasonically dispersed evenly, and then, the solid samples were obtained via high speed centrifugation and vacuum drying. Solid samples were heated for 3 h under 650 °C in an atmosphere of argon.

### 2.3. Characterization

The microstructure of the products was determined using a scanning electron microscope (SEM, Hitachi, Tokyo, Japan) and transmission electron microscope (TEM, FEI Tecbau G2 F30, Hillsboro, OR, USA, 300 kV). The X-ray diffractometer (XRD, PANalytical MPD, Oak Ridge, TN, USA) test utilizes Cu Kα as the radiation source, with an incident wavelength of λ = 0.15418 A, a current of 40 mA, tube pressure at 40 kV, a sweep speed of 5° min^−1^, and a scanning range set from 5° to 80°. X-ray photoelectron spectroscopy (XPS): a Mono X-ray source with a beam spot size of 500 µm and an energy of Al-Kα (hv = 1486.6 ev) was utilized. The instrument employed was the ESCALab250-Xi model, Thermo Fisher Scientific Co., Ltd., Waltham, MA, USA, with an energy step set at 0.050 eV. Raman spectra was examined at a range of 100–2000 cm^−1^ under a 325 nm He-Ne laser source using a LabRAM Aramis spectrometer, Bagnols sur Cèze, France. This work innovatively used TG-FTIR-GCMS technology to characterize the carbonization process of purified diatom in real time. This integral combination can be used to observe the concrete changes of the solid phase and gas phase in carbonization in real time. TG-DSC was carried out from room temperature to 650 °C at a rate of 10 °C min^−1^. FTIR spectra were obtained under the range of 500–4000 cm^−1^. Gas chromatography mass spectrometry (GCMS) was examined respectively at 400 °C, 500 °C, and 600 °C.

### 2.4. Cell Fabrication and Electrochemical Analysis

The electrochemical performance of the PD@C anode was evaluated via coin-type cells (CR2032) assembled in the glovebox (Ar-filled). The active materials (PD@C), super P, and polyvinylidene difluoride (PVDF) were directly mixed with a mass ratio of 7:2:1 and then evenly dispersed in the N-methyl-2-pyrrolidine (electronic-grade NMP).

The slurry was cast to the surface of copper foil and dried for 12 h at 80 °C (vacuum). Then, the dried sheet was sliced into a circular shape with a diameter of 14 mm. Lithium foils were used as the counter electrodes. LiPF_6_ was dissolved in a mixed solution of EC/DEC/DMC with a volume ratio of 1:1:1. Meanwhile, microporous polypropylene Celgard 2500 was used as the separator.

Cyclic voltammetry (CV) curves were collected over a voltage range of 0.01–1.5 V (vs Li/Li^+^). The measurements of electrochemical impedance spectroscopy (EIS) were carried out at a frequency range from 10 mHz to 1 MHz with an AC amplitude of 5 mV using an electrochemical workstation (CHI 660B). The cycling tests of galvanostatic charge–discharge were achieved on a multichannel battery analyzer (LAND test system). All parameters were tested under room temperature.

### 2.5. Theoretical Calculations

First-principles calculations were based on density functional theory (DFT). The DFT calculations were determined via the Cambridge Sequential Total Energy Package (CASTEP), which is derived from pseudopotential plane waves (PPW). For electron–electron interaction and exchange potential, generalized gradient approximation (GGA) and Perdew–Burke–Ernzerhof (PBE) potential energy principles were used. Among which, the energy cutoff plane fluctuation energy is 480 eV, and the electron self-consistent field tolerance rate is 2 × 10^−6^ eV/atom. In the geometric optimization, all atomic positions were allowed to relax, and the atoms were optimized until the threshold force was less than 0.05 eVÅ^−1^. Brillouin areas were calculated using Monkhorst–Pack (MP) grid sampling.

The adsorptive binding energies (*E_ads_*) were defined as:

(1) $$E_{ads}=E_{total}-E_{s}-E_{a}$$

*E_total_* is the total system energy of Li^+^ on different electrode surfaces (SiO_2_, C, and SiO_2_@C); *E_a_* and *E_s_* are the adsorption energies of Li^+^ and the substrate, respectively.

## 3. Results and Discussion

**Figure 1:**
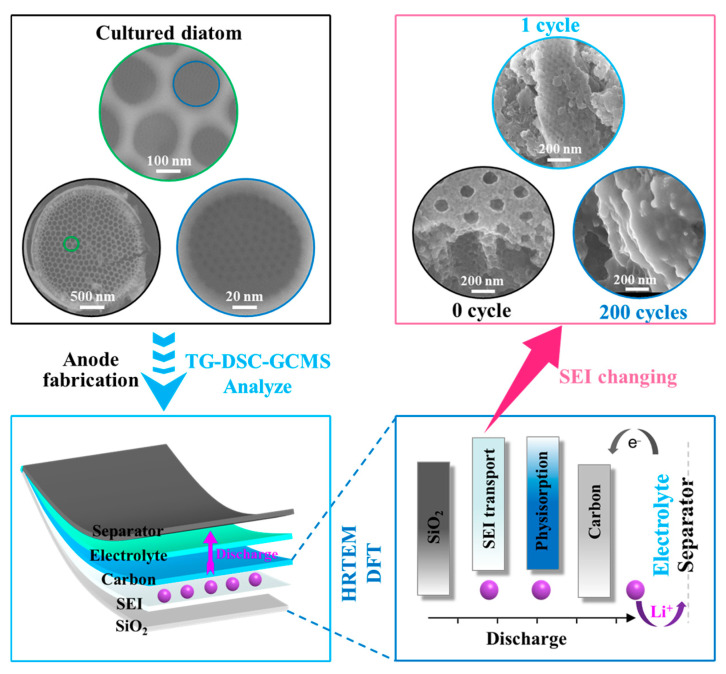
Schematic illustration of characterization of diatom biomass and their applications as lithium anodes.

The main research pathway of the paper is illustrated in Figure 1. The research concept aims to develop anodes for LIBs using cultured diatoms. By employing innovative TG-FTIR-GCMS technology, real-time determination of the primary elements and products involved in carbonization is achieved, along with simultaneous analysis of the main components of the remaining substances. Analysis of cycled anodes clarifies the transport modes of Li^+^, including SEI transport and physisorption. General microscopic characterization methods are utilized for SEI transport, while DFT calculations are employed for physisorption.

### 3.1. Characterization of the PD@C Composites

#### 3.1.1. The Formation Process of Carbon Layer

After undergoing a series of washes with hydrochloric acid, absolute ethanol, and deionized water, the washed liquid became transparent and colorless (Figure S1). Subsequently, after the carbonization process, the original samples changed from pale green powder to black powder (PD@C composites, Figure S2), and the concentration of various metal elements in the sintered sample decreased to its lowest levels. Specifically, the removal of Al 0.21%, Ca 0.02%, Fe 0.05%, K 0.05%, Mg 0.01%, and Na 0.02% was evident compared to the initial sample. The corresponding results are depicted in Figure S3.

**Figure 2:**
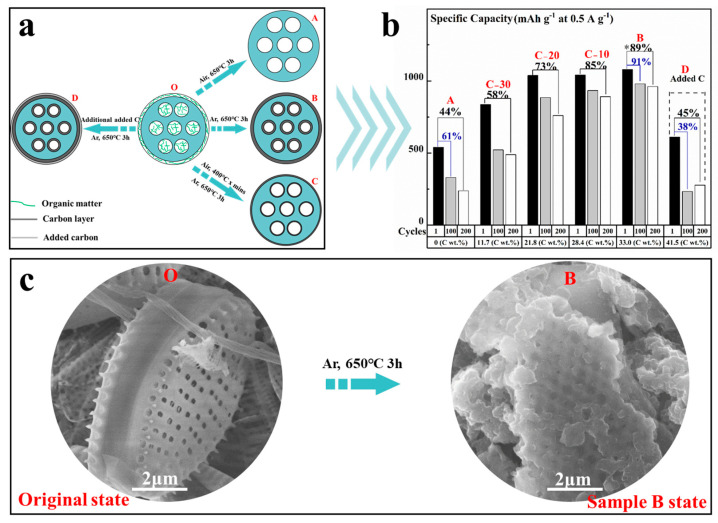
(**a**) Manufacturing process of carbon layer, and (**b**) capacity of different carbon content after different cycles. (**c**) SEM comparison figures of purified diatoms and processed purified diatoms with carbon content of 33 wt.%. * Optimum electrochemical performance.

In the experiments, the primary synthesis routes were focused on preserving the varying carbon content by adjusting calcinating conditions to achieve optimal properties. Different manufacturing processes (Figure 2a) resulted in different capacity outcomes (Figure 2b). PD@C-A (C: 0 wt.%) exhibited an initial charge capacity of ~530 mAh g^−1^ with a capacity retention ratio of only 44% after 200 cycles. In contrast, PD@C-B (C: 33 wt.%) demonstrated excellent initial charge capacity and a high capacity retention ratio of 1080 mAh g^−1^ and 89%, respectively, by effectively utilizing the inherent carbon source. On the other hand, external introduction of carbon through a precursor method increased the carbon content of PD@C-D to 41.5 wt.% but led to significantly reduced performance, with an initial charge capacity and capacity retention ratio after 100 cycles at only 611 mAh g^−1^ and 38%. This indicates that enhancing anode performance does not rely on externally introduced carbon but rather on maximizing the utilization of intrinsic organic matter. The outer carbon coating on diatom frustules was unable to fully penetrate their multistage nanopores, leading to inferior performance compared to PD@C-B despite different levels of carbon content, as shown in Figure 2b. SEM comparison images in Figure 2c further illustrate the effect of the carbon layer coating on purified diatoms and PD@C-B.

**Figure 3:**
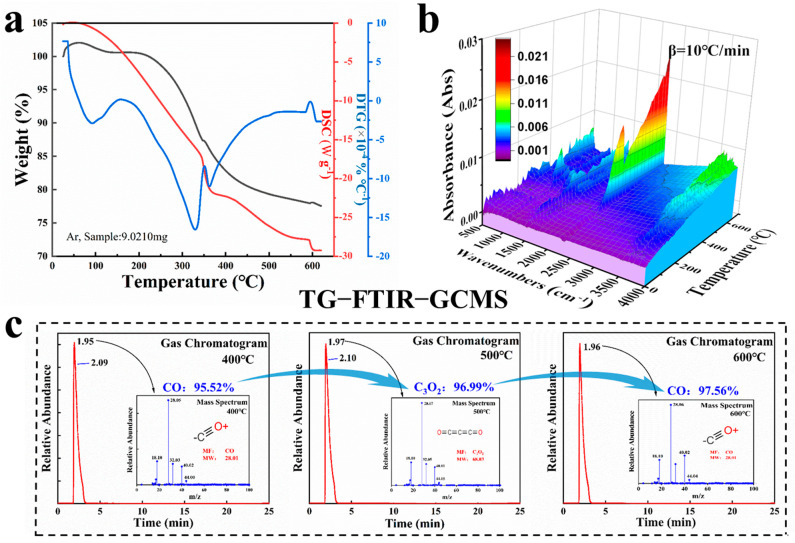
TG−FTIR−GCMS results of the process in the carbonization: (**a**) TG−DSC curves, (**b**) 3D TG−FTIR spectra, and (**c**) chromatogram of GC−MS to identify the emitting compounds; refer to Figure S4 for further MS information.

In this study, TG-FTIR-GCMS was utilized for real-time analysis of the carbonization process. The thermogravimetric results of the PDMs are presented in Figure 3a, depicting three distinct stages. The first phase (room temperature to 130 °C) corresponds to the complete evaporation of residual moisture from the PDMs. The second phase (200 to 600 °C) is associated with the combustion and carbonization of organic substances in the PDMs, resulting in a weight reduction of approximately 23%. In the third phase (600 to 650 °C), minimal weight loss is observed as remaining substances stabilize. Additionally, FTIR analysis from 500 to 4000 cm^−1^ was conducted throughout the heating process (Figure 3b). Analysis of the data revealed: (I) slight enhancement in stretching vibration of the H-O group at peaks of 3500–3700 cm^−1^ with increasing temperature; gradual increase in the peak at ~2250 cm^−1^ related to C≡N; and changing characteristic peaks at ~1600 cm^−1^ corresponding to C=C and ~1450 cm^−1^ corresponding to C-O-C, indicating hemicellulose formation [28]. (II) Spectra for PD@C materials exhibited a SiO_2_ component at the peak of the ~1000 cm^−1^ region (Si-O-Si), and there was noticeable appearance of a peak in the 550 cm^−1^ region with increasing temperature, indicating asymmetric bending and stretching vibrations of SiO bands, suggesting reduction of part of SiO_2_ to SiO by C on the surface of the diatom frustule, which can significantly enhance the electrical conductivity of the diatom cell.

Finally, the simultaneous experiments of GCMS were examined at different heating stages (Figure 3c); refer to Figure S4 for further MS information. During the calcination process at 400 °C, combustion of residual organic matter occurred, resulting in the emission of gases containing CO (peak area: 95.52%), C_4_N_4_Ni_4_ (1.34%), N_2_ (0.25%), C_3_O_2_ (0.19%), CH_3_F_2_N (0.17%), C_3_H_4_ (0.13%), and others. Similarly, at 500 °C, the emitted gases contained components such as C_3_O_2_ (96.99%), C_23_H_30_O_4_ (1.47%), C_2_H_2_N_2_O (0.62%), CO (0.45%), C_3_H_4_ (0.14%), and C _5_H_3_N_3_ (0.13%). At 600 °C, the components were CO (97.56%), C_4_H_4_O (1.2%), C_3_H_4_ (0.51%), C_2_H_2_N_2_O (0.28%), C_3_H_4_N_2_ (0.25%), and C_3_O_2_ (0.15%); refer to Table S3 for more details. The GCMS data obtained at 400 °C, 500 °C, and 600 °C indicate that the chemical reactions during carbonization of PDMs mainly involve two elements—carbon and oxygen—with the main emitted products being CO and C_3_O_2_. The results also reveal a higher content of carbon compared to other elements in the residual organic matter of PDMs.

#### 3.1.2. The Composition Analysis of Composites

**Figure 4:**
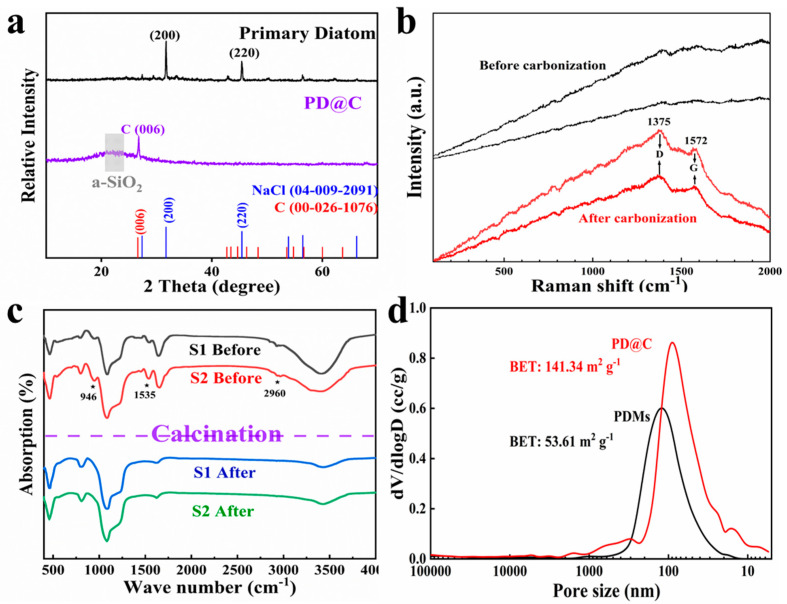
Test results of sample before and after carbonization: (**a**) XRD, (**b**) Raman, (**c**) FTIR, and (**d**) BET.

Figure 4a exhibits the XRD pattern of the PD@C composites. The broad peak at 22° corresponds to the amorphous SiO_2_ phase, and the peak of 26.6° can be assigned to the carbon phase [29] in PD@C. Furthermore, the XRD comparison results between PD@C and primary diatom indicate that the primary samples underwent a removal process of inorganic salts (such as NaCl, AlCl_3_, etc) during treatment. Additionally, two characteristic peaks at ~1375 cm^−1^ and ~1572 cm^−1^ correspond to the D-band and G-band of carbon, respectively (Figure 4b), which are caused by the vibrations of sp^2^ and sp^3^ carbon [29,30]. Moreover, the Raman result of the reference sample (before carbonization) exhibited no discernible D-band and G-bond. Comparison of the infrared spectra before and after carbonization (Figure 4c) indicates that some bonds in the PDMs have broken and converted into gas (CO, C_3_O_2_), consistent with previous findings [31,32]. According to the BET tests (Figure 4d), the specific surface area of PD@C reached 141.34 m^2^ g^−1^, significantly higher than its pre-carbonization value of 53.61 m^2^ g^−1^; larger pores were caused by etching. The BET adsorption curve and pore release associated with the treatment process are depicted in Figure S5.

**Figure 5:**
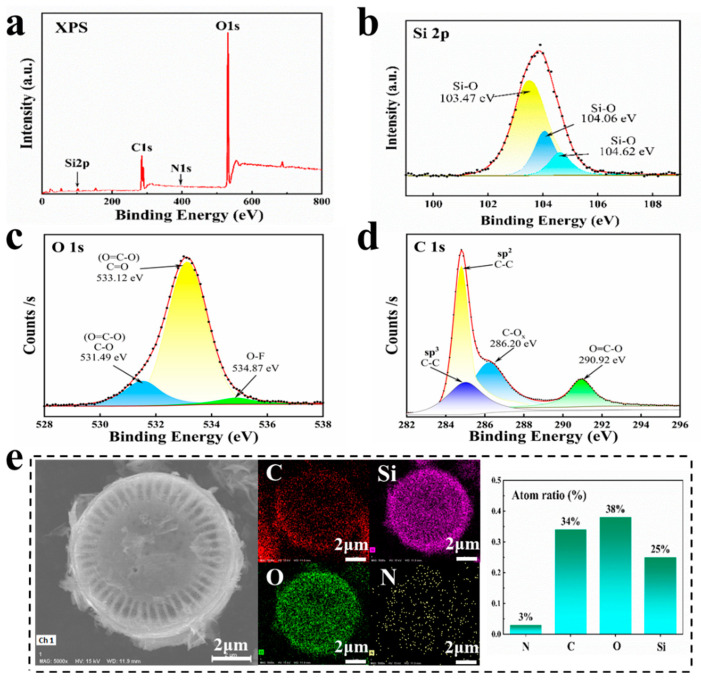
Test results of sample after carbonization: (**a**–**d**) XPS, (**e**) SEM-EDS.

Furthermore, in combination with XPS and EDS, the elemental analysis of PD@C active material was conducted. Figure 5a displays the XPS peaks of the PD@C sample, confirming that it is primarily composed of elements such as Si, O, C, and N. The Si 2p high-resolution spectrum (Figure 5b) reveals a Si-O peak at 103.4 eV, indicating that silicon exists mainly in the form of SiO_2_ accompanied by SiO (Si-O peak at 104.6 eV). Additionally, Figure 5c shows peaks at 531.5 eV and 533.1 eV in the O 1s spectrum, suggesting that O exists predominantly in the forms of O=C-O bond [33], while Figure 5d demonstrates a peak at 284.8 eV in the C 1s spectrum, confirming that carbon is primarily deposited as elemental layers with the additional presence of either C=N or C-O_x_ bonds (peak at 286.2 eV), as well as O=C-O bonds (peak at 290.9 eV). Furthermore, combined with SEM-EDS elemental results (Figure 5e), it was found that Si, O, C not only constitutes the main existing element but also exhibits uniform distribution within diatom structures. Although the nitrogen content is low (Figure S6), its distribution remains relatively uniform. According to EDS atom ratio test results, the ratio of oxygen atoms to silicon atoms is determined to be approximately 1:0. This confirms that part of the SiO_2_ on the diatom shell surface has been reduced to SiO via carbon deposition. Furthermore, the proportion of carbon atoms after explicit deposition is approximately 34%.

### 3.2. Anode Application and Its Electrochemical Characterization

**Figure 6:**
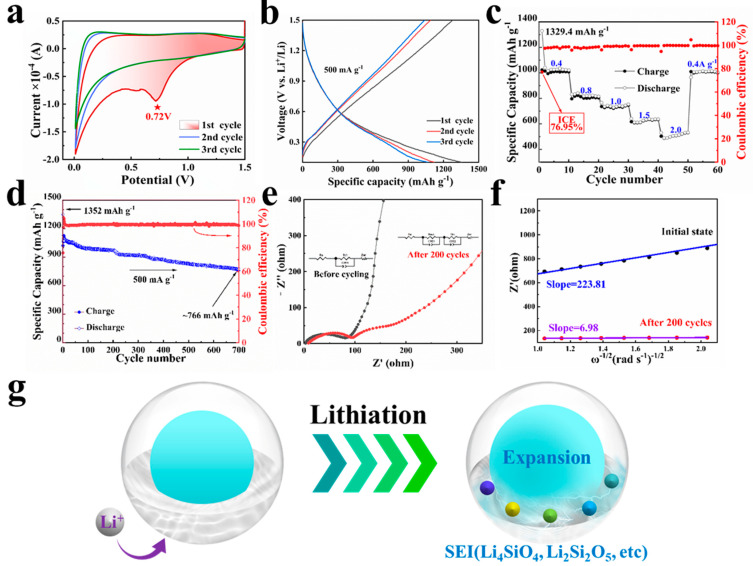
Electrochemical performance of the PD@C anode: (**a**) CV curves, (**b**) selected galvanostatic charge/discharge profiles, (**c**) rate ratio, (**d**) cycling performance, (**e**,**f**) EIS and the curves of Z’ and ω^−1/2^, and (**g**) schematic illustration of the bonds formed between the ${\mathrm{SiO}}_{x}$ shell and the lithiation product in sufficient electrical contact.

A series of electrochemical tests were conducted by fabricating half-cells to evaluate the performance of the PD@C anode. Initially, CV and rate performance tests were employed to elucidate the working mechanism of the anode. The CV curve in the first cycle of the PD@C anode distinctly exhibits a reduction peak at 0.72 V (Figure 6a), which is attributed to the formation of the solid electrolyte interface (SEI) film. Additionally, galvanostatic charge/discharge profiles of the first three cycles indicate that continuous slopes are consistent with characteristics of the SiO_2_-based anode (Figure 6b). A schematic illustration depicts bonds formed between the shell and lithiation product providing sufficient electrical contact (Figure 6g), while conversional processes involving reactions between SiO_x_, Si, and Li^+^ are represented by following equations (Equations (2)–(5)) [34]. Furthermore, absence of a reduction peak in the 2nd and 3rd curves indicates that the initial structure layer of SEI film has been formed in the 1st cycle, and overlapping subsequent curves represent the reversibility of the lithiation reaction. Moreover, the rate performance test was utilized to investigate the effect on the electrochemical properties related to the continuous porous structure of the PD@C composite (Figure 6c). The PD@C anode displayed a high initial irreversible capacity of 1329.4 mAh g^−1^ with an ICE of 76.95%, which is higher than the reported Si-based anodes. The PD@C anode showed capacities of 1023, 797, 736, 616, and 502 mAh g^−1^ at the current densities of 0.4, 0.8, 1.0, 1.5, and 2.0 A g^−1^, respectively. Herein, the outstanding long-term cycle performance means the PD@C anode has solved the volume expansion of the Si-based anode very well (Figure 6d).


(2) $${\mathrm{SiO}}_{x}+2x{\mathrm{Li}}^{+}+2xe^{-}\to x{\mathrm{Li}}_{2}O+\mathrm{Si}$$

(3) $$4{\mathrm{SiO}}_{x}+4x{\mathrm{Li}}^{+}+4xe^{-}\to x{\mathrm{Li}}_{4}{\mathrm{SiO}}_{4}+(4-x)\mathrm{Si}$$

(4) $$5{\mathrm{SiO}}_{x}+2x{\mathrm{Li}}^{+}+2xe^{-}⇌x{\mathrm{Li}}_{2}{\mathrm{Si}}_{2}O_{5}+(5-2x)\mathrm{Si}$$

(5) $$\mathrm{Si}+x{\mathrm{Li}}^{+}+xe^{-}⇌{\mathrm{Li}}_{x}\mathrm{Si}\;(\mathrm{Alloying reaction of Si})$$

In addition, EIS was used to elucide the Li^+^ diffusion kinetics and impedance variation of the battery. As shown in Figure 6e, the charge transfer resistance of the PD@C anode is 93.84 Ω after 200 cycles, which is 2.23% higher than that of the initial anode. The results exhibit the excellent stability of the electrode structure. Meanwhile, based on the simulated equivalent circuit (Figure 6e, inset), a stable SEI film (R_SEI_: 42.09 Ω) was formed at the interface of the PD@C anode after 200 cycles. The fitted Z’ vs. ω^−1/2^ in the high frequency region decreased sharply from 223.81 (before cycle) to 6.98 (after 200 cycles), as shown in Figure 6f. According to Equation (6), the Li^+^ diffusion coefficient was 1.03 × 10^−12^ cm^2^ s^−1^, which was almost one thousand times higher than before (1.01 × 10^−15^ cm^2^ s^−1^). The results reveal that the cycled anode has a faster Li^+^ transport rate and faster kinetics.

(6) $$D_{Li^{+}}=\frac{R^{2}T^{2}}{2A^{2}n^{4}F^{4}c^{2}\delta^{2}}$$

**Figure 7:**
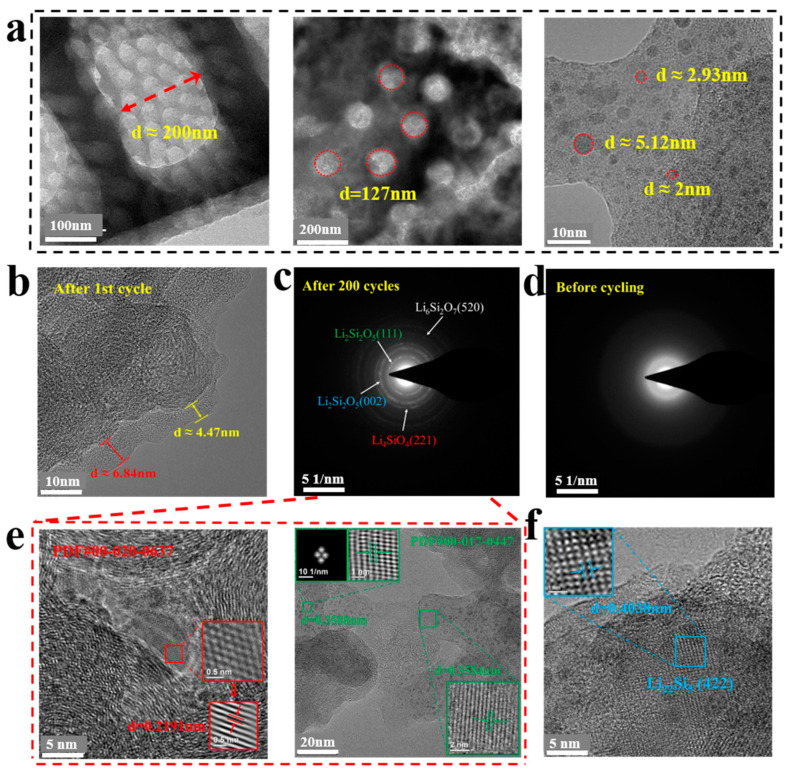
(**a**) Macropores, mesopores, and micropores of the PD@C via TEM, (**b**) SEI layer formed after the first cycle, TEM-SAED pattern of the anode (**c**) after 200 cycles and (**d**) before cycling, and (**e**,**f**) TEM images of the anode after 200 cycles.

Moreover, the electrochemical behavior of the PD@C electrode sheet was analyzed by integrating the microscopic morphology and chemical composition of the electrode material before and after charging and discharging. Firstly, the hierarchical porous substructure of the PD@C was analyzed using TEM. Figure 7a depicts the structure with macropores of around 200 nm, mesopores of around 120 nm, and micropores in the range of 2–5 nm. Additionally, after the first cycling, the SEI film measured 4–7 nm, as shown in Figure 7b. The amorphous state of the primary PD@C was confirmed through the TEM-SAED pattern (Figure 7d), while lattice fringes were observed in the SAED pattern of the anode after 200 cycles (Figure 7c). Furthermore, TEM results after 200 cycles in Figure 7e indicated the presence of Li_4_SiO_4_, Li_2_Si_2_O_5_, and Li_6_Si_2_O_7_ phases in the SEI layer. Lastly, a small portion of Li–Si alloy was observed to exist in the form of Li_22_Si_5_ (Figure 7f).

### 3.3. Theoretical Calculations of C Layer Enhancement Effect

**Figure 8:**
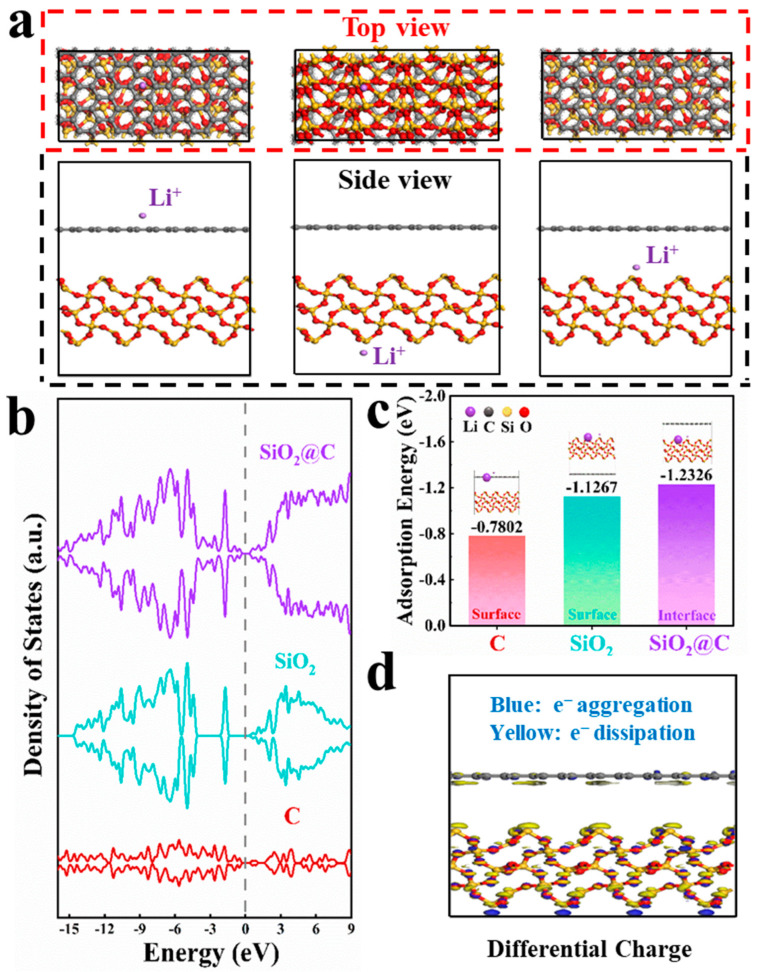
(**a**) Side view and top view of three sites of Li^+^ adsorption, (**b**) density of states of C, SiO_2_, and SiO_2_@C, (**c**) adsorption energies of different positions, and (**d**) differential charge results of the SiO_2_@C.

DFT calculations were carried out to unveil the outstanding electrochemical properties of the PD@C samples. The structure model of the PD@C composite was established by stacking SiO_2_ and C, with the crystal plane of SiO_2_ being (100) and that of C being (002). Initially, the adsorption capacity of different components for Li^+^ was simulated and calculated. The density of states of C, SiO_2_, and SiO_2_@C in Figure 8b indicates that SiO_2_@C composites combine graphite characteristics and exhibit excellent electron penetration at the Fermi level. Particularly, SiO_2_@C demonstrates electronic state growth near the Fermi level compared to pure SiO_2_. Consequently, the graphite layer enhances electrical conductivity, promotes electrochemical reaction kinetics, and provides more charge storage sites. Furthermore, in the differential charge results for SiO_2_@C (Figure 8d), blue represents electron aggregation, while yellow represents electron dissipation. These differences visually indicate that there is no net charge gain or loss at the interface of SiO_2_@C, suggesting Van der Waals forces rather than stable chemical bonds between SiO_2_ and C. However, there is some coupling between interfaces leading to a slight charge transfer. This accumulation contributes to improved conductivity as well.

The possible adsorption sites and adsorption energy of Li^+^ in SiO_2_@C were investigated to simulate the real sites of Li^+^ storage in the electrochemistry process. Figure 8a shows the side view and top view of three sites of Li^+^ adsorption. The adsorption energies of the C surface, SiO_2_ surface, and SiO_2_@C are −0.7802 eV, −1.1267 eV, and −1.2326 eV, respectively (Figure 8c), indicating that the intermediate position of C and SiO_2_ is more favorable for Li^+^ adsorption, which is consistent with the electrochemical lithium storage position of SiO_2_. The above results demonstrate that the SiO_2_@C anode has the following advantages: (I) The carbon coating not only enhances electrical conductivity but also provides adsorbable Li^+^ active sites at the interface of the composite (in addition to the chemical storage of lithium by SiO_2_). (II) The adsorption energy of Li^+^ facilitates its embedding in the interface of SiO_2_@C, thereby promoting the transport kinetics of Li^+^ and accelerating the reaction rate.

**Figure 9:**
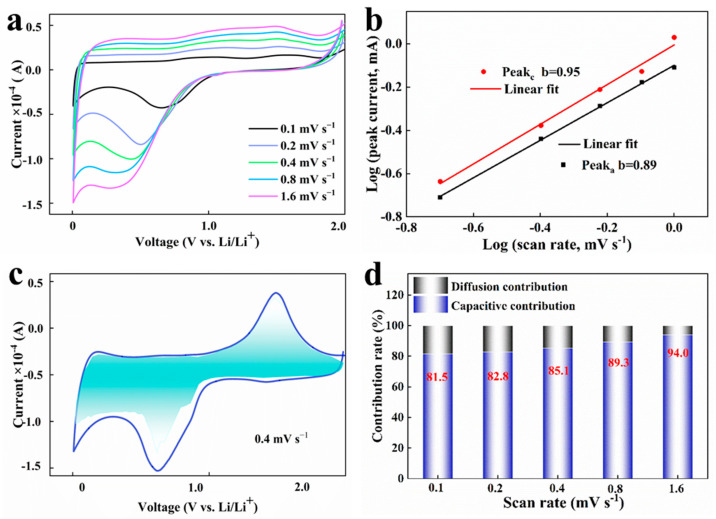
(**a**) CV curves of PD @ C electrode materials at different sweeping rates, (**b**) fitting diagram of sweep speed and current, (**c**) proportion of pseudocapacitance at a sweep rate of 0.4 mV s^−1^, (**d**) contributions of volume control and diffusion control at different scan rates.

To verify the conclusion of the DFT calculation, the capacity control contribution and diffusion control contribution were fitted by measuring the first lap CV at different scan rates in the range of 0.1–1.6 mV s^−1^. Figure 9a shows that the PD@C electrode still exhibits a distinct redox peak current with different scan rates and covers a wider potential range. The current at different scan rates is fitted using Formula:


(7) $$i=av^{b}$$

In the formula, *a* and *b* are fitting parameters. *i* represents the peak current, and the peak current of the sample at various scan rates can be determined from the graph, with the peak current and sweep speed further calculated using formula:


(8) logi=loga+blogv

When the value of b approaches 0.5, it indicates that the electrochemical reaction is primarily governed by the diffusion process. When the value of b approaches 1, it suggests that the electrochemical reaction is predominantly influenced by the capacitance process. The fitting slope b for Peakc of the PD@C electrode is 0.95 and for Peaka is 0.89 (Figure 9b), indicating that the lithium storage behavior of this electrode material is controlled by a pseudocapacitance process. The relative contribution of the diffusion and capacitance processes can be determined using the following formula:

(9) $$i(V)=k_{1}v+k_{2}v^{1/2}$$

(10) $$i(V)/v^{1/2}=k_{1}v^{1/2}+k_{2}$$

Among it, $k_{1}v$ represents the contribution of capacity control, while $k_{2}v^{1/2}$ represents the contribution of diffusion control, and the Equations (7)–(10) are based on a pertinent study [35]. Refer to Figure 9c for the fitting diagram at a scan rate of 0.4 mV s^−1^. The contribution of the capacity control increases with the scan rate, as shown in Figure 9d, with values of 81.5%, 82.8%, 85.1%, 89.3%, and 94.0% at scan rates of 0.1 mV s^−1^, 0.2 mV s^−1^, 0.4 mV s^−1^, 0.8 mV s^−1^, and 1.6 mV s^−1^, respectively.

To sum up, the part of the reversible chemical reaction in the PD@C electrode material is the primary contributor to over 80% of the capacity. The contribution ratio of pseudocapacitance increases with the scan rates during the cycles, indicating that chemical reversible reactions dominate lithium storage behavior. Additionally, the carbon layer in PD@C provides more electrochemical active sites for accelerated electron transport and contributes about ten percent to the diffusion control capacity, corresponding to the previous DFT calculations.

## 4. Conclusions

In summary, this study employed a biomass diatom-based anode for LIBs and provided a detailed demonstration of their electrochemical performance and working mechanism. The excellent electrochemical properties of the PD@C anode were primarily attributed to the hierarchical porous structures and intrinsic biomass carbon content (33 wt.%) of the diatoms. During carbonization, the main emissions were CO and C_3_O_2_ from organic combustion, resulting in PD@C mainly consisting of SiO_2_, amorphous C (sp^2^, sp^3^), and SiO. Furthermore, analysis of the working mechanism revealed that the SEI layer was composed of Li_4_SiO_4_, Li_2_Si_2_O_5_, Li_6_Si_2_O_7_, and Li–Si alloy. The PD@C composite also exhibited a physisorption capacity for Li^+^, with DFT calculations indicating the highest adsorption energy (−1.2326 eV) at the intermediate position between C and SiO_2_. These results suggest that carbon coating not only enhances electrical conductivity but also provides active sites for adsorbable Li^+^. Overall, this work lays a foundation for developing advanced biomass-based SiO_x_ anodes as well as utilizing primary biomass organic matter in value-added applications.

## Data Availability

The original contributions presented in the study are included in the article/Supplementary Material, further inquiries can be directed to the corresponding author.

## Supplementary Materials

The following supporting information can be downloaded at: https://www.mdpi.com/article/10.3390/ma17184473/s1, Figure S1: The washing process of the raw inactivated diatoms; Figure S2: Images of PDMs and PD@C; Figure S3: The concentration of various metal elements in the sintered samples; Figure S4: The chromatogram of MS to identify the emitting compounds; Figure S5: The BET adsorption curve and pore release during the treatment process; Figure S6: The SEM-EDS elemental results; Table S1: The utilization of natural diatoms in the realm of functional materials; Table S2: The comparison of electrochemical properties; Table S3: The data of GCMS at 400 °C/500 °C/600 °C during calcination; Table S4: Abbreviation table. References [20,36,37,38,39,40,41,42,43,44,45,46,47,48,49,50,51,52] are cited in the Supplementary Materials.
