# Supplementary material for: Diatom-Based Artificial Anode—Uniform Coating of Intrinsic Carbon to Enhance Lithium Storage
Source: Materials (Basel). 2024 Sep 12;17(18):4473. doi: 10.3390/ma17184473 (PMC11433294; doi:10.3390/ma17184473)
Supplement: Supplementary file 1 [file materials-17-04473-s001.zip › materials-3186094-supplementary.pdf]

Support Information

Diatom-Based Artificial Anode－Uniform Coating of Intrinsic Carbon to Enhance Lithium Storage

Table S1 The utilization of natural diatoms in the realm of functional materials

Diatom species	Application fields	Ref.
Phaeodactylum tricornutum	Nano-silver composite material	[36]
Coscinodiscus wailesii	Biosensor	[37]
Aulacoseira sp.	Biosensor	[38]
Eolimna minima	Nano-gold composites	[39]
Nitzschia palea	Photovoltaic material	[40]
Thalassiosira pseudonana	Drug carrier	[41]
Thalassiosira weissflogii	Silicone silica composition	[42]
Coscinodiscus sp. , Coscinodiscus wailesii	Light capture material	[43]
Pinnularia sp.	Nano-TiO_2 composites	[44]

Table S2 The comparison of electrochemical properties

Material	ICE (%)	Specific Capacity (mA h g^{-1})	Ref
Miled SiO_2	37	800 (100 cycles at 100 mA g^{-1})	[45]
CS-SG	59	560 (30 cycles at 50 mA g^{-1})	[46]
SiO_2/TiO_2/Ppy	55	433 (50 cycles at 44 mA g^{-1})	[47]
N-OMC/SiO_2	51	630 (100 cycles at 200 mA g^{-1})	[48]
BP-CS	62.6	611 (200 cycles at 200 mA g^{-1})	[20]
Ni/SiO_2	56.6	672 (50 cycles at 100 mA g^{-1})	[49]
SiO_2/TiO_2@C	66	998 (100 cycles at 100 mA g^{-1})	[50]
SiO_2/MXene	70	820 (100 cycles at 200 mA g^{-1})	[51]
SiO_2/C	46	600 (50 cycles at 200 mA g^{-1})	[52]
PD@C	77	924 (200 cycles at 400 mA g^{-1})	This work

Table S3

The data of GCMS at 400 ℃ during calcination			
No.	CAS<br>(matching with detected)	Name	Peak Area (%)

1	630-08-0	CO	95.52
2	13463-39-3	$C_4O_4Ni_4$	1.34
3	7440-37-1	Ar	0.49
4	7727-37-9	N_2	0.25
5	53593-70-7	$C_8H_2O_5V$	0.23
6	504-64-3	C_3O_2	0.19
7	753-58-2	CH_3F_2N	0.17
8	74-99-7	C_3H_4	0.13
9	105492-44-2	$C_{17}H_{15}NO_3$	0.12
10	927-74-2	C_4H_6O	0.1
**The data of GCMS at 500 °C during calcination**			
1	504-64-3	C_3O_2	96.99
2	23925-70-4	$C_{23}H_{30}O_4$	1.47
3	288-37-9	$C_2H_2N_2O$	0.62
4	630-08-0	CO	0.45
5	74-99-7	C_3H_4	0.14
6	39805-98-6	$C_5H_3N_3$	0.13
**The data of GCMS at 600 °C during calcination**			
1	630-08-0	CO	97.56
2	1119-19-3	C_4H_4O	1.2
3	74-99-7	C_3H_4	0.51
4	288-37-9	$C_2H_2N_2O$	0.28
5	33898-53-2	$C_3H_4N_2$	0.25
6	504-64-3	C_3O_2	0.15

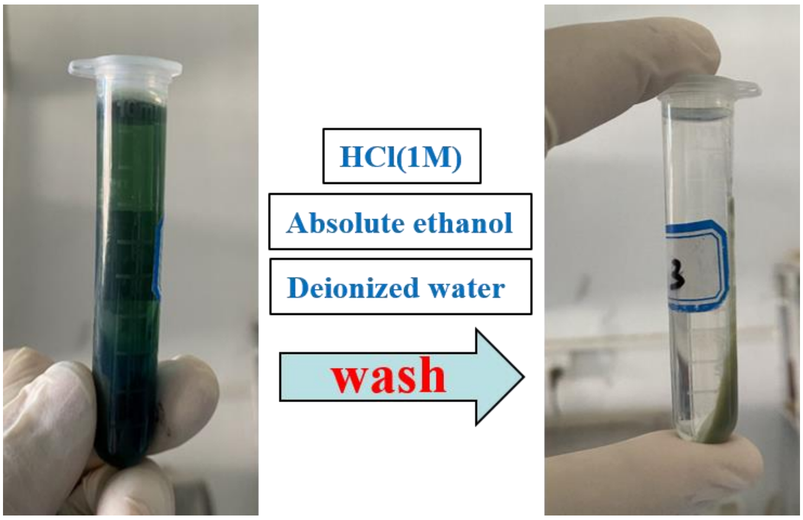

Fig. S1 The washing process of the raw inactivated diatoms.

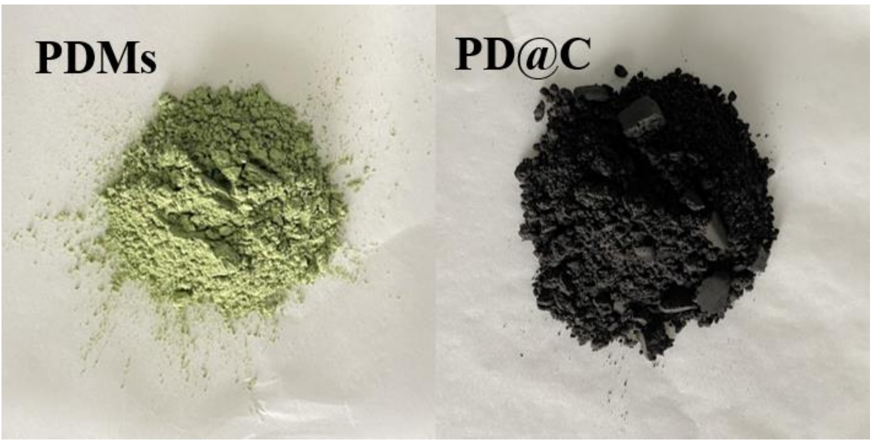

Fig. S2 Images of PDMs and PD@C.

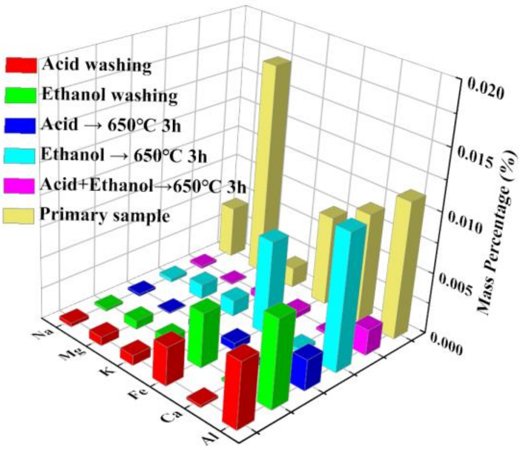

Fig. S3 The concentration of various metal elements in the sintered samples

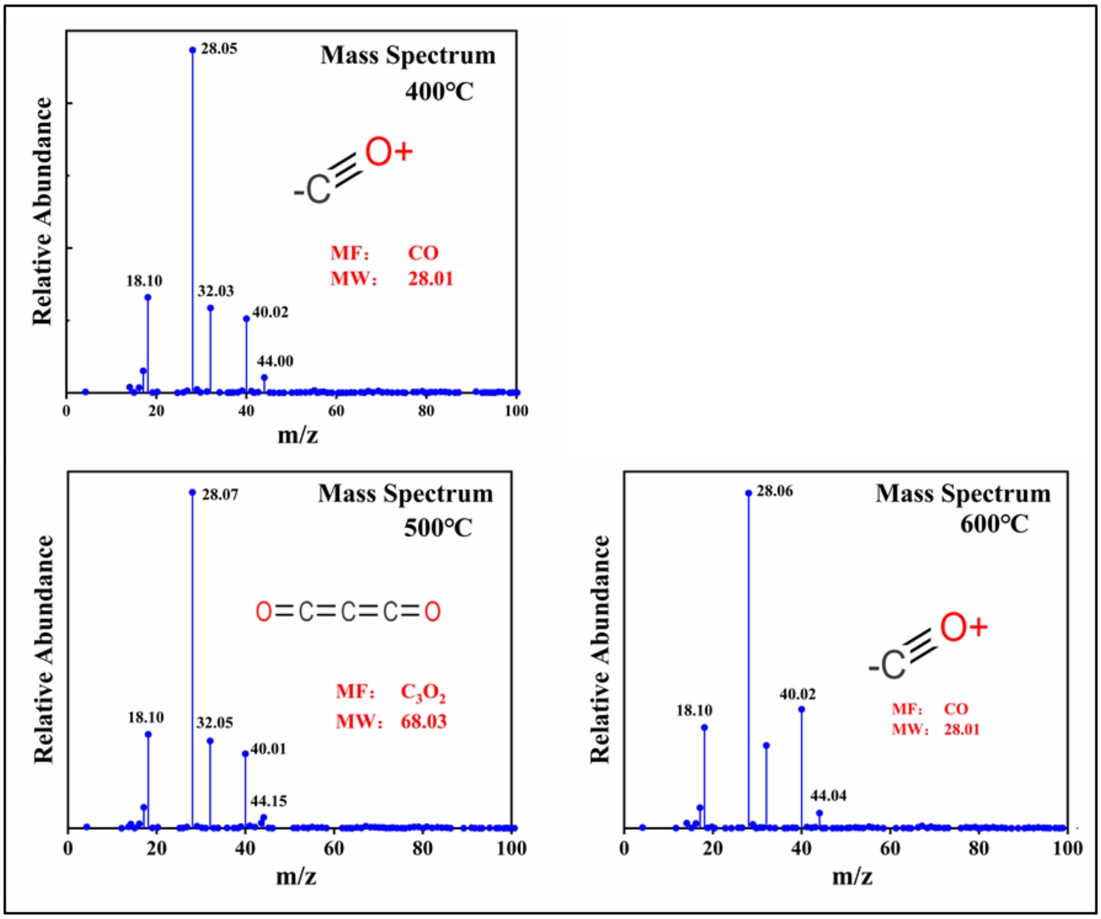

Fig. S4 The chromatogram of MS to identify the emitting compounds.

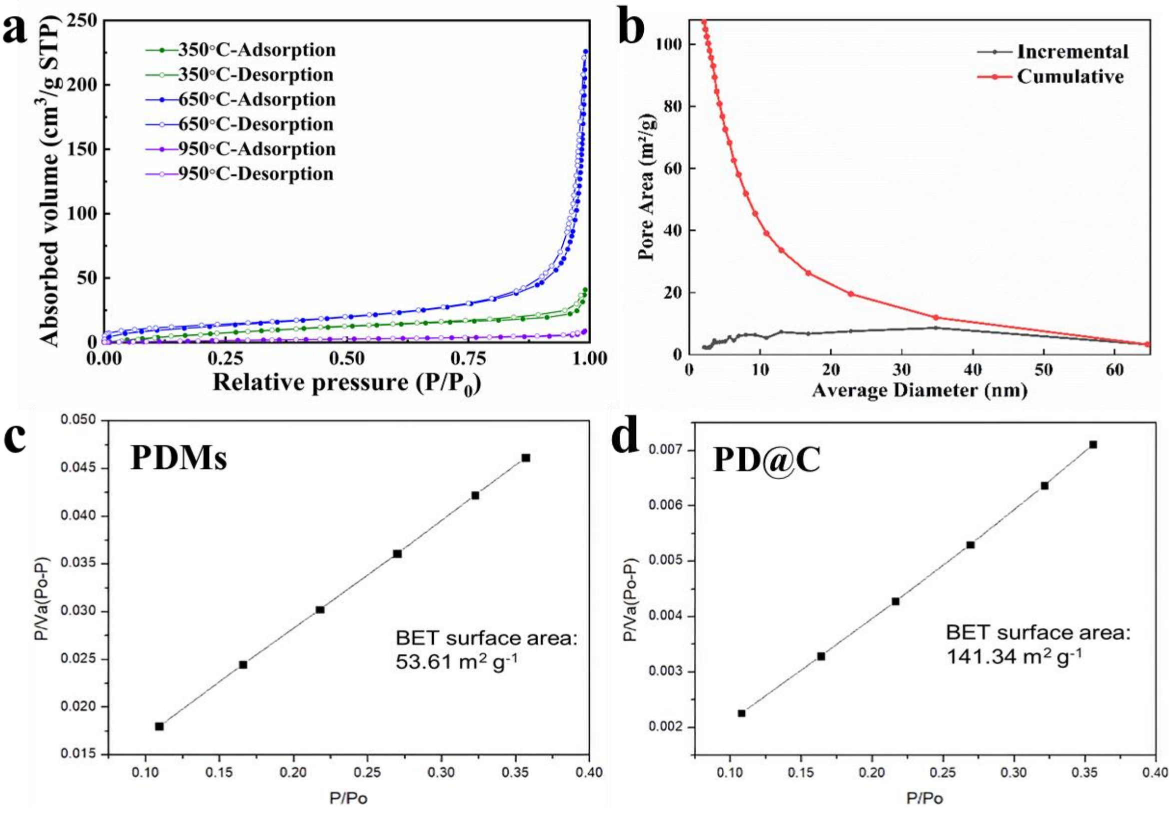

Fig. S5 The BET adsorption curve and pore release during the treatment process.

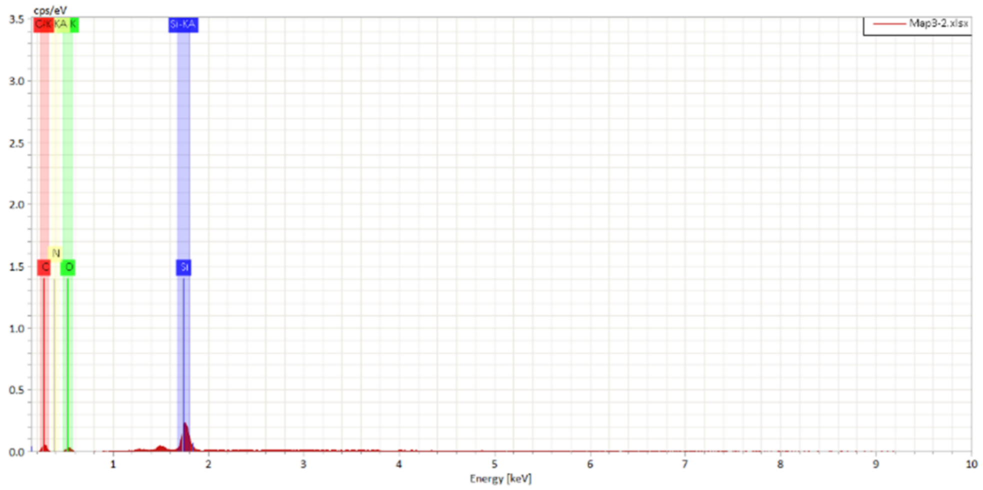

Fig. S6 The SEM-EDS elemental results.

Table S4 Abbreviation table

English name	Abbreviation
Purified Diatom	PD
Processed Diatom and Carbon	PD@C
Ethylene Carbonate	EC

Dimethyl Carbonate	DMC
Ethyl Methyl Carbonate	EMC
Solid Electrolyte Interface	SEI
N-methylpyrrolidone	NMP
Polyvinylidene Fluoride	PVDF
Three-dimensional	3D
Initial Coulomb Efficiency	ICE
Cyclic Voltammetry	CV
Electrochemical Impedance Spectroscopy	EIS
Scanning Electron Microscope	SEM
Transmission Electron Microscope	TEM
High Resolution Transmission Electron Microscope	HRTEM
Selected Area Electron Diffraction	SAED
Energy Dispersive Spectrometer	EDS
X-ray Diffractometer	XRD
X-ray Photoelectron Spectroscopy	XPS
Thermogravimetry coupled with Infrared Spectroscopy and Gas Chromatography Mass Spectrometry	TG-FTIR-GCMS
Density Functional Theory	DFT
